# Impairment of *Drosophila* Orthologs of the Human Orphan Protein C19orf12 Induces Bang Sensitivity and Neurodegeneration

**DOI:** 10.1371/journal.pone.0089439

**Published:** 2014-02-21

**Authors:** Arcangela Iuso, Ody C. M. Sibon, Matteo Gorza, Katharina Heim, Cristina Organisti, Thomas Meitinger, Holger Prokisch

**Affiliations:** 1 Institute of Human Genetics, Technische Universität München, Munich, Germany; 2 Institute of Human Genetics, Helmholtz Zentrum München, Neuherberg, Germany; 3 Department of Cell Biology, University Medical Center Groningen, University of Groningen, Groningen, The Netherlands; 4 Sensory Neurogenetics Research Group, Max Planck Institute of Neurobiology, Martinsried, Germany; Louisiana State University Health Sciences Center, United States of America

## Abstract

Mutations in the orphan gene *C19orf12* were identified as a genetic cause in a subgroup of patients with NBIA, a neurodegenerative disorder characterized by deposits of iron in the basal ganglia. C19orf12 was shown to be localized in mitochondria, however, nothing is known about its activity and no functional link exists to the clinical phenotype of the patients. This situation led us to investigate the effects of *C19orf12* down-regulation in the model organism *Drosophila melanogaster*. Two genes are present in *D. melanogaster*, which are orthologs of *C19orf12*, *CG3740* and *CG11671*. Here we provide evidence that transgenic flies with impaired C19orf12 homologs reflect the neurodegenerative phenotype and represent a valid tool to further analyze the pathomechanism in C19orf12-associated NBIA.

## Introduction

NBIA refers to a heterogeneous group of rare (1–3 cases/1 million population) inherited neurological disorders whose hallmark is iron deposition in the basal ganglia. Currently, nine different subtypes of NBIA have been recognized and associated to their genetic counterpart. *PANK2*, *PLA2G6* and *C19orf12* are hot spot genes, with *C19orf12* accounting alone for up to 30% of cases. Most of the knowledge about *C19orf12*-associated NBIA refers to genetic, clinical and radiological features. So far, 67 patients have been reported in literature [1]–[9] but still no hypothesis has been made concerning the pathomechanism underlying this complex disorder. Two postmortem brain samples were examined and showed iron accumulation, Lewy bodies, tau pathology and spheroids with a strong immunoreactivity for ubiquitin. However, the scarcity of cases makes it difficult to state whether the overlap between C19orf12-associated NBIA and Parkinson’s disease is a rule or a private trait.

*C19orf12* expression occurs mainly in brain, blood cells and adipocytes. During *in vitro* adipocyte differentiation *C19orf12* expression increases constantly and is co-regulated with genes involved in the fatty acid metabolism suggesting a role for C19orf12 in lipid metabolism [1].

C19orf12 harbors two trans-membrane domains. Immunofluorescence experiments have shown that C19orf12 is localized in mitochondria. Based on these evidences, MPAN (Mitochondrial membrane Protein-Associated Neurodegeneration) has been proposed as acronym to refer to *C19orf12*-associated NBIA. Although C19orf12 is a mitochondrial protein, mutations in *C19orf12* do not appear to affect the mitochondrial bioenergetics in fibroblasts under basal conditions [1].

The scarcity of data available from neuropathology, the limitations of investigating the biochemical basis of the disease directly in the brain, the absence of a phenotype in fibroblasts and the lack of homology to proteins with known function, led us to work on the creation of a model that would allow us to study the consequences of impaired C19orf12 in tissues which are primarily affected. We decided to set up a *D. melanogaster* model. *Drosophila* was successfully employed to study pantothenate kinase-associated neurodegeneration (PKAN) resulting from mutations in *PANK2* [10], [11] and tested in rescue experiments with pantethine [12]. We identified two *D. melanogaster* orthologs of *C19orf12*, *CG3740* and *CG11671.* We used double heterozygous deletion flies and an RNAi approach to down-regulate the expression of both genes providing evidence that the orthologs are required for the maintenance of the brain tissue and the normal locomotor function. Our results suggest that *Drosophila* can be used to gain further insight in MPAN disorder.

## Results and Discussion

### *Drosophila* has Two Orthologs of the Human *C19orf12*

**Figure 1:**
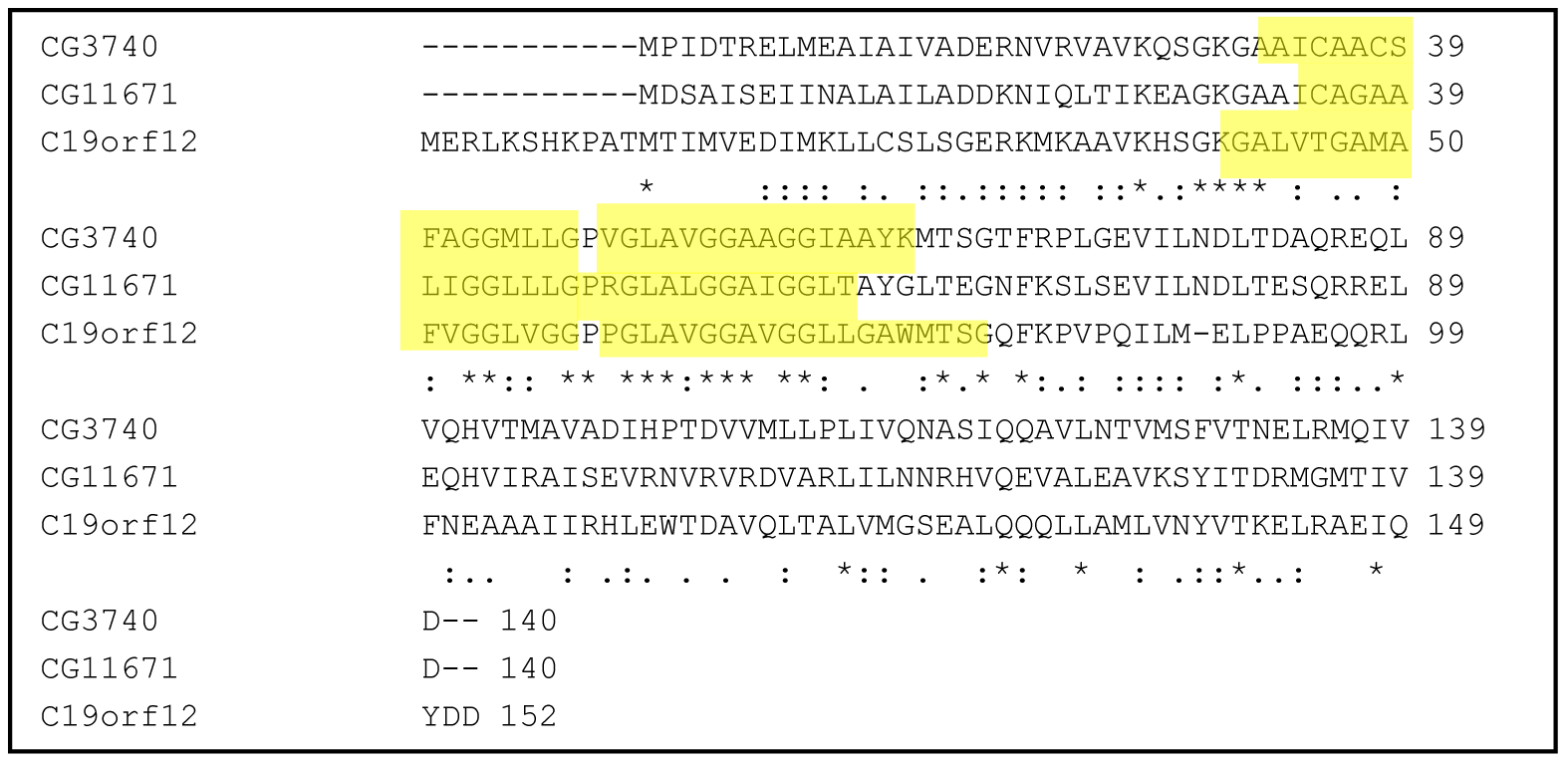
Alignment of the fly C19orf12 orthologs (CG3740, CG11671) with human C19orf12 protein. Proteins have been aligned using Clustal 2.1 multiple alignment tool. Stars (*) indicate identities and dots indicate a higher (:) and a lower (.) degree of similarity. The two *D. melanogaster* proteins are 63% and 55% similar to C19orf12 and share 72% similarity with each other. The transmembrane domains predicted by PolyPhobius are marked in yellow.

**Figure 2:**
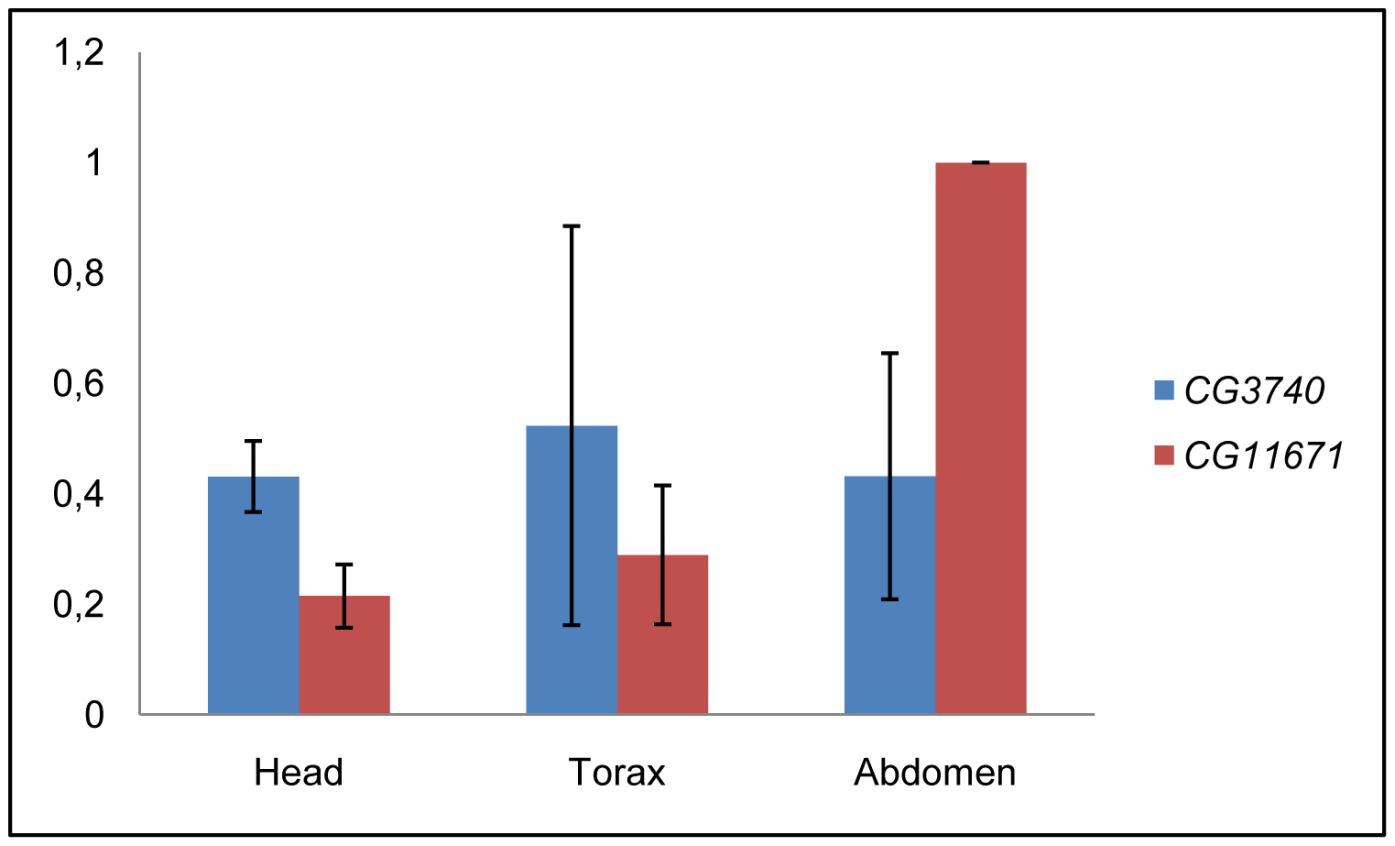
Expression of *CG3740* and *CG11671* in head, thorax and abdomen of *D. melanogaster*. The analysis has been performed on total RNA extracted from adult w^1118^ flies using the same number of males and females (n = 4). The *Ribosomal Protein 49* has been used as endogenous control to normalize *CG3740* and *CG11671* expression level. The level of *CG11671* in the abdomen has been set to 1 and the expression of *CG3740* and *CG11671* in the other tissues expressed relatively.

Comparative genomics revealed that the human *C19orf12* gene has two orthologs in *D. melanogaster*, *CG3740* and *CG11671,* which share 63% and 55%, respectively, of similarity at protein level with C19orf12 (NP_001026896.2) (Figure 1). *CG3740* is localized on the X-chromosome in position 2B5–2B5 while *CG11671* maps to chromosome 3R in position 84F8–84F8. The two *Drosophila* genes are proposed to originate by a duplication event at the speciation node explaining their 72% similarity. The transmembrane domain is the only feature of C19orf12 that is predicted by the primary amino acidic sequence. PolyPhobius program [13] predicted two transmembrane domains for CG3740, similar to the human protein, and one long one for CG11671. Aside the prediction, it is also possible that CG11671 has two transmembrane domains which would also fit with the length of the predicted domain (Figure 1). *CG3740* and *CG11671* are differentially expressed (Figure 2). The analysis of the mRNA expression pattern in head, thorax and abdomen of adult w^1118^ flies revealed that expression levels of *CG3740* are comparable among the tissues tested, while *CG11671* expression is low in head and high in abdomen.

### Generation of Double RNAi and Double Heterozygous Deletion Flies

**Figure 3:**
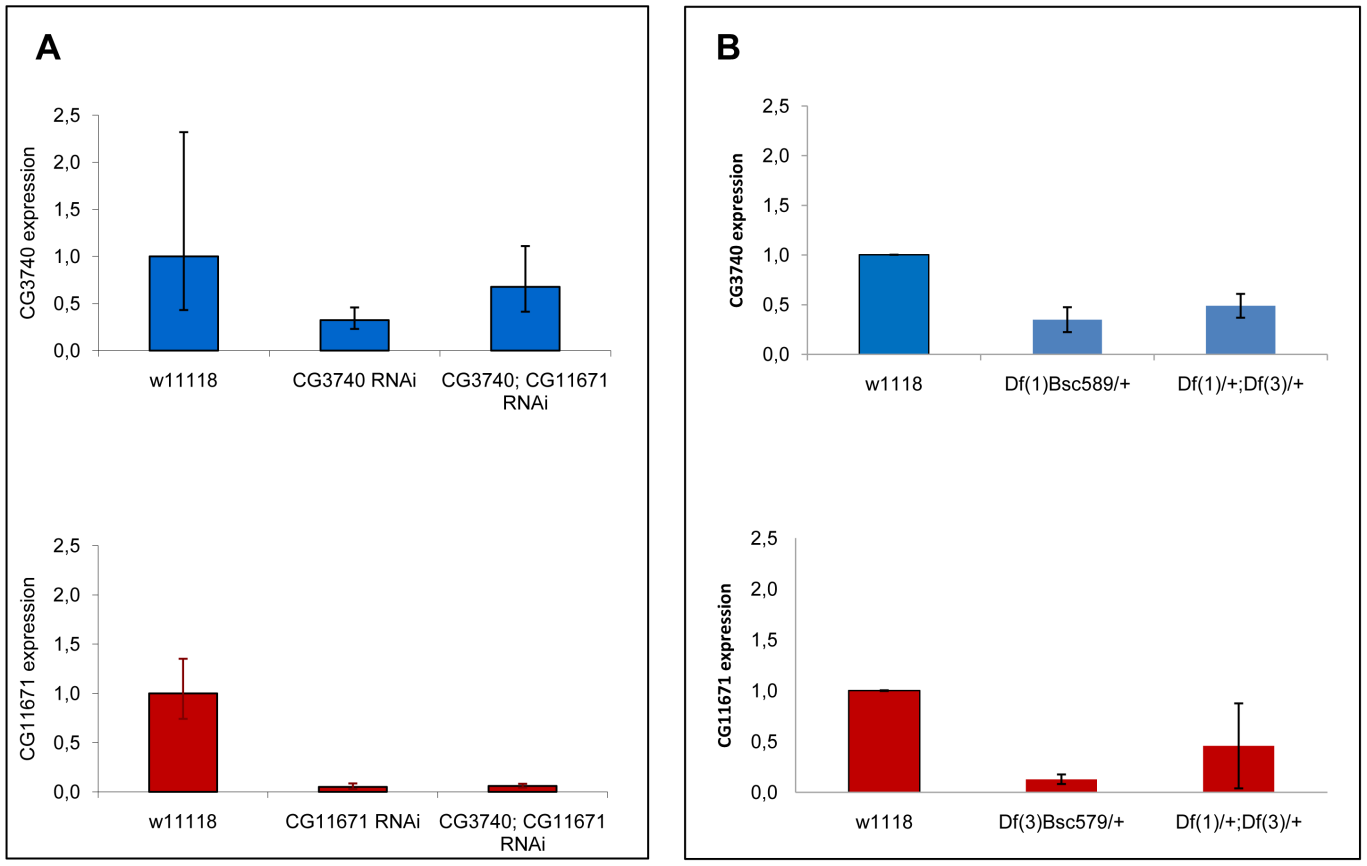
Expression of *CG3740* and *CG11671* in heads from 10 days-old flies. The *Ribosomal Protein 49* has been used as endogenous control to normalize *CG3740* and *CG11671* expression level. (**A**) *CG3740* and *CG11671* have been measured in single RNAi flies (*CG3740* RNAi and *CG11671* RNAi) and in double RNAi flies (*CG3740*; *CG11671* RNAi) using the same number of males and females (n = 4). The level of *CG3740* and *CG11671* in control flies has been set to 1 and the expression of both genes in down-regulated flies expressed relatively. (**B**) *CG3740* and *CG11671* have been measured in single heterozygous deletion flies (*Df(1)*BSC589/+ and *Df(3L)*BSC579/+) and in double heterozygous deletion flies (*Df(1)*BSC589/+; *Df(3L)*BSC579/+) using female flies (n = 4). The level of *CG3740* and *CG11671* in control flies has been set to 1 and the expression of both genes in heterozygous deletion flies expressed relatively. Data refer to the average of three independent experiments ± SD.

In order to analyze the consequences of impaired activity we made use of the bipartite GAL4-UAS system. Transgenic lines carrying the yeast Upstream Activating Sequence (UAS) in front of the RNAi constructs for both *CG3740* and *CG11671* were available at the Vienna *Drosophila* RNAi stock collection [14]. Both RNAi lines were separately crossed with specific driver lines expressing GAL4 under the control of *elav*, *tubulin* or *actin* promoters, enabling knockdown of *CG3740* and *CG11671* gene products at the neuronal level (*elav*) or in the whole body (*actin*, *tubulin*). Quantitative PCR performed in RNA isolated from heads of 10 days-old flies revealed that the strongest knockdown was induced under the *actin* promotor expression, leaving mRNAs levels of only 25% for *CG3740* or 10% for *CG11671* (Figure 3A). However, down-regulation of the product of each single gene did not result in any significant difference in lifespan or climbing activity compared to control flies (data not shown).

Based on the homology between *CG3740* and *CG11671* and the overlapping expression pattern, we hypothesized a compensative mechanism between the two genes that can justify the absence of any pathological phenotype. Hence we generated a stock carrying both RNAi transgenes and crossed these flies with the driver line *act*-GAL4 in order to reduce compensatory effects between *CG3740* and *CG11671* (Figure S1). Quantitative PCRs performed on heads of 10 days-old flies showed a reduction of at least 40% for *CG3740* and 75% for *CG11671* in double RNAi transgenic flies (Figure 3A).

In a second approach, heterozygous deletion strains for *CG3740* and *CG11671* were generated to validate RNAi fly phenotypes. For this purpose, w^1118^ flies were crossed with *Df(1)*BSC589 and *Df(3)*BSC579 flies, two lines carrying macro deletions encompassing *CG3740* and *CG11671* respectively and several other genes. Moreover, *Df(1)*BSC589 and *Df(3)*BSC579 lines were crossed between each other in order to generate double heterozygous deletion flies (Figure S2). All heterozygous deletion flies were morphologically normal. Quantitative PCRs performed showed expression levels comparable to RNAi transgenes (Figure 3B).

### Double RNAi Flies Have Reduced Median Lifespan

**Figure 4:**
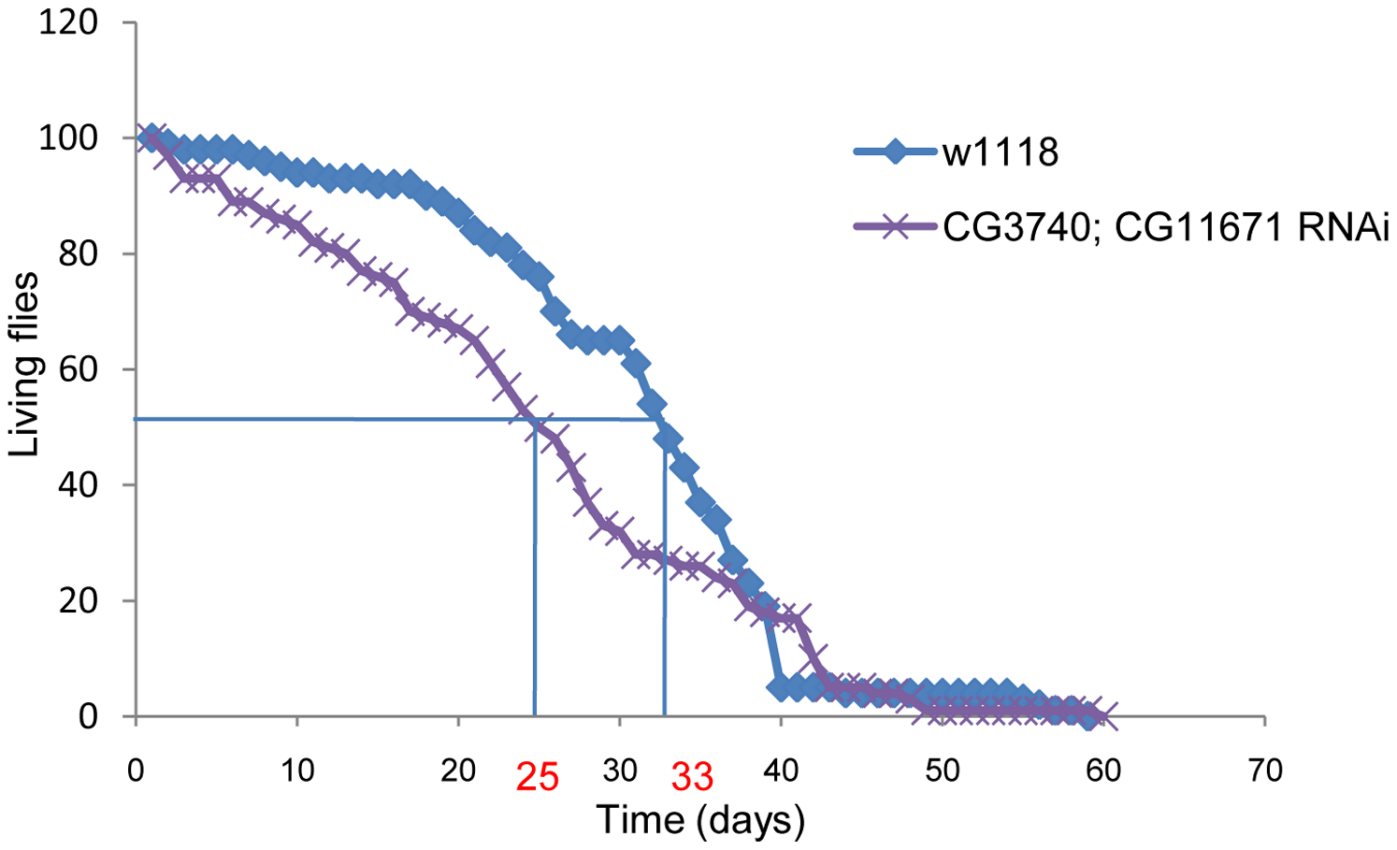
Kaplan-Maier curve of control (w^1118^) and double RNAi male flies (n = 100). The median lifespan is 33 days for control flies vs. 25 days for down-regulated flies. The difference was statistically evaluated by Log Rank test (p = 0.02).

The lifespan of control w^1118^ and double RNAi flies was determined under standard growth conditions by counting the number of dead flies every second day. Down-regulated flies revealed a significantly reduced median lifespan of about 8 days at 50% survival (Figure 4).

### Double RNAi and Double Heterozygous Deletion Flies Show Defective Climbing

**Figure 5:**
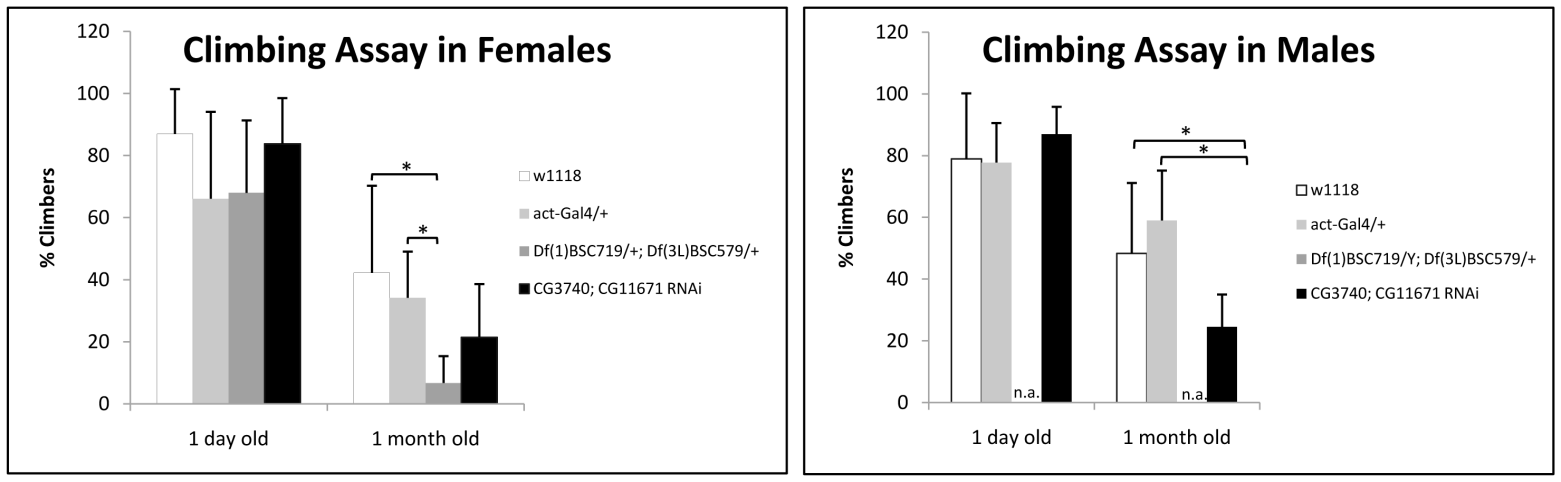
Climbing activity in young (1 day-old) and aged (1 month-old) control (w^1118^ and *act*-GAL4/+), double RNAi and double heterozygous flies. The average is given for 50 animals in each group.

NBIA patients with defective C19orf12 display spasticity and progressive cognitive decline. Onset generally occurs in childhood up to early adulthood with slow progression and survival well into adulthood [1], [15]. Although double RNAi and double deletion flies are able to walk, climb and fly, we noticed that they recovered more slowly after the application of a force by tapping them to the lower part of the vial. Those flies able to recover and climb were slower than the controls. We quantified the behavioral defects. Since locomotion can be affected by the age, we tested flies of two different ages using age-matched controls for all tests. In addition, two different control strains were used in the assay, the canonical wild-type w^1118^ and the *act*-GAL4/+ flies obtained by backcrossing *act*-GAL4 for 6 generations with w^1118^ (Text S1). When analyzing young flies no differences between double RNAi, double deletion and control flies were observed. However, 4-weeks-old double RNAi and double deletion flies performed poorly in the climbing assay compared to controls (Figure 5). Only few down-regulated flies were able to reach the end point fixed at the 150 ml line (17.5 cm) of a cylinder in fifteen seconds. The majority only initiated climbing and stopped before the endpoint was reached, fell back or did not initiate climbing. This behavior suggested a neuromuscular phenotype in double RNAi and double heterozygous deletion flies.

### Double RNAi Flies Present Vacuoles in the Brain

**Figure 6:**
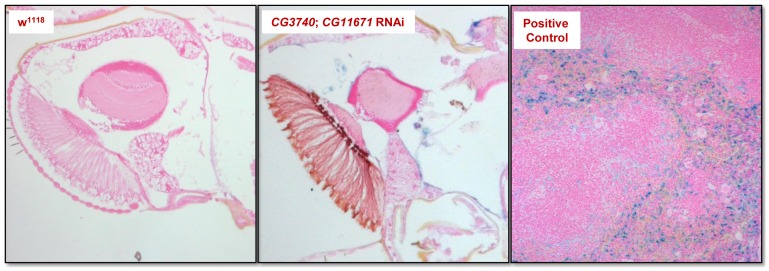
Prussian blue staining for detection of iron deposits. The staining was performed on paraffin-embedded sections of 28 days-old flies. No iron deposits were detected in the medulla or retina of either controls or double RNAi flies. Clear iron inclusions were present in paraffin-embedded sections of mouse spleen (positive control).

Since the hallmark of NBIA is iron accumulation in the brain, we searched for iron accumulation in fly brains of double RNAi flies. The presence of iron deposits was investigated using the standard Prussian blue staining on paraffin-embedded brains of aged flies using sections of mouse spleen as a positive control for the staining. Under our experimental conditions we could not find iron deposits (Figure 6).

**Figure 7:**
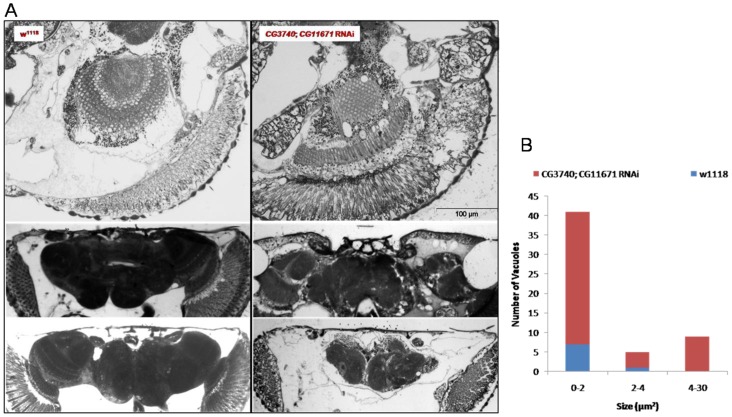
Detection of vacuoles in ultrathin Epon plastic sections. A) Horizontal head sections from 28 days-old control (w^1118^) and down-regulated (*CG3740*; *CG11671* RNAi) flies. B) Number and size of vacuoles have been quantified with Image J running the applications “Find Edges” and “Analyze Particles” on thresholded 8-bit pictures. Particle size was imposed bigger than 20 pixels^2^ and with a circularity factor between 0,5 and 1. Detected vacuoles were displayed using “Overlay Mask”. Data were manually validated to exclude artifacts. Brains analyzed for each fly strain: n = 3. Scale bar: 100 µm.

We morphologically analyzed brains for signs of neurodegeneration in order to investigate whether the defective climbing coincided with abnormalities in the nervous system. In *Drosophila*, the tissue loss is typically detected as accumulation of degenerative vacuoles or holes in the brain [16], [17]. For this we analyzed the morphology of brains from 28 days-old double RNAi and age-matched control flies. A high number of vacuoles with an area up to 30 µm^2^ was found in the brain and in the optical lobe of all investigated down-regulated flies suggesting a neurodegenerative process. Few small vacuoles below 2 µm^2^ were also found in one control fly as a normal result of the aging process [18] (Figure 7A–B).

### Double RNAi and Double Heterozygous Deletion Flies Present Bang Sensitivity

**Figure 8:**
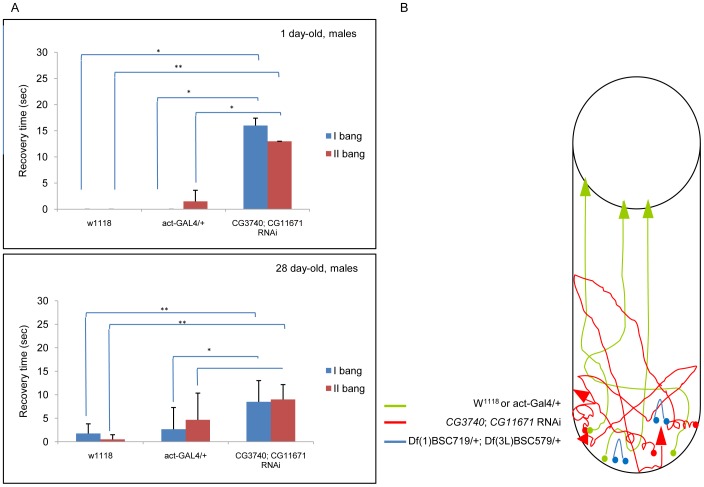
Bang test in young (1 day-old) and aged (1 month-old) control (w^1118^ and *act*-GAL4/+) and double RNAi males. A) Flies have been vortexed twice (Bang I and II) with 10 minutes in between and the time needed to upright recorded. A) Representative trajectories are reported for aged control (green), double RNAi (red) and for double heterozygous (blue) flies.

In order to further analyze possible locomotor defects, we subjected young and adult double RNAi and double heterozygous flies to mechanical stress by vortexing them shortly and banging the vial against a hard surface. Often, a compromised mitochondrial metabolism generates bang sensitive mutants which respond to the mechanical boost with a temporary paralysis [19]. Control flies restored the upright position instantly and climbed the vial (Movie S1; Movie S2). Double RNAi flies failed to recover a correct body position promptly even if no signs of paralysis or failure of movements were present (Movie S3; Movie S4). They frantically moved their legs and bodies and jumped repeatedly from one side to the other of the vial before getting the upright position and climbing the tube. Double heterozygous deletion flies actually restored the upright position instantly, like controls, but ceased moving. No flies climbed up (Figure 8; Movies S1, S2, S3, and S4).

## Concluding Remarks

*Drosophila* models have been successfully applied as a tool to unravel basic mechanisms underlying a large number of human neurodegenerative disorders such as Parkinson’s disease [20], Alzheimer [21], [22], Non-coding Trinucleotide Repeat diseases [23] and PKAN [10], [11]. Here we provide compelling evidence that an *in vivo* model for MPAN has been generated by down-regulating the two homologues of *C19orf12*. The uncoordinated behavior or lack of orientation in the bang test, the impaired climbing activity as well as the shorter lifespan and the morphological alterations in brain suggest that double RNAi flies can be used as a model for MPAN. The presence of locomotor impairment and bang sensitivity also in double heterozygous deletion flies provides additional genetic evidence that the phenotypes detected in double RNAi flies are due to the knock-down of *CG3740* and *CG11671* rather than due to off-target effects. The availability of *CG3470*; *CG11671* down-regulated flies provide affected tissues, like brain or muscles which are the main targets of NBIA disorders but seldom available for functional studies, and may represent a valid tool for the screening of therapeutics.

## Materials and Methods

### Drosophila Genetics

Fly stocks were maintained routinely in our lab at 22°C according to standard protocols. As wild type stock w^1118^ and *act*-GAL4/+ were used. UAS-*CG3740* (FBst0477282), UAS-*CG11671* (FBst0463955) transgenic lines were obtained from Vienna Drosophila RNAi Center (Vienna, Austria); w^1118^ (BL5905), *act5C*-GAL4/CyO (BL4414), CyO/sna^Sco^ (BL2555), CyO/sna^Sco^; TM6B/MKRS (BL2551), *Df(1)*BSC589 w^1118^/FM7h/Dp(2;Y)G, P{hs-hid}Y (BL25423), w^1118^; *Df(3L)*BSC579/TM6C, cu^1^ Sb^1^ (BL25413) were from Bloomington Drosophila Stock Centre (Indiana University, USA). UAS-*CG3740*/CyO; UAS-*CG11671*/TM6B strain, *Df(1)*BSC589/+, *Df(3L)*BSC579/+ and *Df(1)*BSC589/+; *Df(3L)*BSC579/+ were generated in this work. Chromosomes 1, 3, 4 of the driver line *act5C*-GAL4/CyO were isogenized to the genetic background of control w^1118^ flies by back crossing it for 6 generations. Control *act*-GAL4/+ flies were obtained by back crossing w^1118^ with *act*-GAL4 flies for 6 generations (Text S1).

### RNA Extraction and qPCR

Flies were cut in three parts: head, thorax and abdomen. Four specimens from each part were collected, resuspended in 500 µl of Trizol (Invitrogen) and homogenized using a teflon pestle. Total RNA was isolated using the Trizol method. One microgram of RNA was reversely transcribed using the M-MLV Reverse Transcriptase (Promega) and oligo dT. *CG3740* and *CG11671* expression were evaluated in comparison to the ribosomal protein 49. The following primers were used:

CG3740_F: 5′ GGCTGTGCTTAACACTGTCAT 3′.

CG3740_R: 5′ CTACACTGATCACAAAGGTTTC 3′.

CG11671_F: 5′ CAGGGCCATATCCGAAGTTA 3′.

CG11671_R: 5′ TGGTCATTCCCATACGATCA 3′.

Rp49_F: 5′ ATCGGTTACGGATCGAACA 3′.

Rp49_R: 5′ ACAATCTCCTTGCGCTTCTT 3′.

DyNAmo Flash SYBR Green qPCR kit (Finzymes) was used for qPCR and the reactions were run on ABI7900HT instrument (Applied Biosystem). qPCRs have been performed in triplicate on RNA isolated each time from 4 flies.

### Behavioral Assays

Two groups of flies, corresponding to “young” (i.e. 24 h after eclosion) and “aged” (i.e. 4 weeks after eclosion) populations of wild-type, double RNAi and double heterozygous deletion flies, were tested in the behavioral assays. Assays were carried out with male and female down-regulated flies and with female double heterozygous deletion flies. Flies were CO_2_-treated and separated into groups with the same number of individuals per vial one day before the test. Hence flies were subjected to the climbing assay and to the bang test.

The climbing assay was performed according to a modified version of Benzer [24]. 50 flies were placed in a 250-ml glass graduated cylinder that was sealed at the top with parafilm to prevent escape. The flies were gently knocked to the bottom of the cylinder and the number of flies able to cross the 150-ml line (17.5 cm) in 15 seconds was recorded.

The test for bang sensitivity was performed as described by Zhang [25]. Flies were vortexed at maximum setting for 10 sec (Bang I) and the time until all flies upright themselves was recorded. To test for refractory period, the flies were vortexed again 10 min later to see if the flies were still bang sensitive (Bang II). For control as well as mutants, 50 flies were tested for each age.

Statistical analyses were performed using Student’s *t*-test. Data are presented as mean ± Standard Deviation.

### Longevity Test

100 males for control and mutant were collected immediately after eclosion. Every 2 or 3 days flies were passed on fresh food and the number of survived flies plotted in an excel graph until their death.

### Brain Histology

Analysis of heads from 28-day-old double RNAi flies and aged matched controls were fixed in 0.1M sodium cacodylate, 2.5% paraformaldehyde, 2.5% gluteraldehyde and embedded in epon. One micrometer horizontal sections were cut for each head. Section from different brain depths were checked for the presence of vacuoles. Sections were stained with 1% toluidine-blue and 1% borax and investigated with an Olympus BX50 light microscope. The number and size of vacuoles was quantified with Image J.

For detection of ferric iron, paraffin embedded sections were incubated for 45 minutes with Prussian blue stain (10% K_4_Fe(CN)_6_: 20% HCl, 1∶1) prior to de-paraffinization. Three washes with water followed and staining was enhanced using DAB following standard procedures. Afterwards, the sections were de-paraffinized using ascending gradients of ethanol to xylene and preparation were mounted in Permount mounting medium (Fisher Scientific) and imaged on a Olympus BX50 light microscope.

## Supporting Information

Figure S1 **Generation of double RNAi Flies.** UAS-CG3740 and UAS-CG11671 flies have been crossed respectively with the double balancer CyO/sna^Sco^; TM6B/MKRS to block any recombination on the second and on the third chromosome. Hence selected parental lines have been crossed between each other in order to get the double balanced stock UAS-CG3740/CyO; UAS-CG11671/TM6. The homozygous stock UAS-CG3740/UAS-CG3740; UAS-CG11671/UAS-CG11671, produced by backcrossing balanced males and females, has been crossed with *act*-GAL4 driver line to get the double RNAi flies.
(TIF)

Figure S2 **Generation of double heterozygous deletion Flies.** Flies carrying deficiencies on chromosomes 1 (BL25423) and 3 (BL25413) have been crossed with wild type flies w^1118^ to generate single heterozygous deletion flies for *CG3740* and *CG11671*. Then deficiency stocks have been crossed between each other in order to get the double heterozygous deleted stock *Df(1)*BSC589/+; *Df(3L)*BSC579/+.
(TIF)

Text S1 **Generation of** ***act*** **-GAL4/+ flies.** *Act5C*-GAL4/CyO (BL4414) and CyO/sna^Sco^ (BL2555) flies, were backcrossed with w^1118^ flies for 6 generations to produce control flies isogenic with w^1118^ for chromosomes 1, 3, and 4.
(DOCX)

Movie S1 **First bang test, bang I, in control w^1118^ flies.** Males and females were contemporary vortexed for 10 seconds at maximum speed and the time required for recovering the upright position recorded.
(MOV)

Movie S2 **Second bang test, bang II, in control w^1118^ flies.** Males and females were contemporary vortexed, for 10 seconds at the maximum speed, 10 minutes after the first bang. The time required to recover the upright position recorded.
(MOV)

Movie S3 **First bang test, bang I, in double RNAi flies.** Males and females were contemporary vortexed for 10 seconds at maximum speed and the time required for recovering the upright position recorded.
(MOV)

Movie S4 **Second bang test, bang II, in double RNAi flies.** Males and females were contemporary vortexed, for 10 seconds at the maximum speed, 10 minutes after the first bang. The time required to recover the upright position recorded.
(MOV)

## References

[1] Hartig MB, Iuso A, Haack T, Kmiec T, Jurkiewicz E, et al (2011) Absence of an orphan mitochondrial protein, c19orf12, causes a distinct clinical subtype of neurodegeneration with brain iron accumulation. Am J Hum Genet 89: 543–550. 21981780 10.1016/j.ajhg.2011.09.007 PMC3188837

[2] Panteghini C, Zorzi G, Venco P, Dusi S, Reale C, et al (2012) C19orf12 and FA2H mutations are rare in Italian patients with neurodegeneration with brain iron accumulation. Semin Pediatr Neurol 19: 75–81. 22704260 10.1016/j.spen.2012.03.006

[3] Hogarth P, Gregory A, Kruer MC, Sanford L, Wagoner W, et al (2013) New NBIA subtype: genetic, clinical, pathologic, and radiographic features of MPAN. Neurology 80: 268–275. 23269600 10.1212/WNL.0b013e31827e07be PMC3589182

[4] Deschauer M, Gaul C, Behrmann C, Prokisch H, Zierz S, et al (2012) C19orf12 mutations in neurodegeneration with brain iron accumulation mimicking juvenile amyotrophic lateral sclerosis. J Neurol 259: 2434–2439. 22584950 10.1007/s00415-012-6521-7

[5] Dogu O, Krebs CE, Kaleagasi H, Demirtas Z, Oksuz N, et al (2012) Rapid disease progression in adult-onset mitochondrial membrane protein-associated neurodegeneration. Clin Genet 84: 350–355. 10.1111/cge.12079 23278385

[6] Dezfouli MA, Alavi A, Rohani M, Rezvani M, Nekuie T, et al (2013) PANK2 and C19orf12 mutations are common causes of neurodegeneration with brain iron accumulation. Mov Disord 28: 228–232. 23166001 10.1002/mds.25271

[7] Goldman JG, Eichenseer SR, Berry-Kravis E, Zimnowodzki S, Gregory A, et al (2013) Clinical features of neurodegeneration with brain iron accumulation due to a C19orf12 gene mutation. Mov Disord 28: 1462–1463. 23494994 10.1002/mds.25410

[8] Schottmann G, Stenzel W, Lutzkendorf S, Schuelke M, Knierim E (2013) A novel frameshift mutation of C19ORF12 causes NBIA4 with cerebellar atrophy and manifests with severe peripheral motor axonal neuropathy. Clin Genet. 10.1111/cge.12137 23521069

[9] Schulte EC, Claussen MC, Jochim A, Haack T, Hartig M, et al (2013) Mitochondrial membrane protein associated neurodegenration: a novel variant of neurodegeneration with brain iron accumulation. Mov Disord 28: 224–227. 23436634 10.1002/mds.25256

[10] Bosveld F, Rana A, van der Wouden PE, Lemstra W, Ritsema M, et al (2008) De novo CoA biosynthesis is required to maintain DNA integrity during development of the Drosophila nervous system. Hum Mol Genet 17: 2058–2069. 18407920 10.1093/hmg/ddn105

[11] Wu Z, Li C, Lv S, Zhou B (2009) Pantothenate kinase-associated neurodegeneration: insights from a Drosophila model. Hum Mol Genet 18: 3659–3672. 19602483 10.1093/hmg/ddp314

[12] Rana A, Seinen E, Siudeja K, Muntendam R, Srinivasan B, et al (2010) Pantethine rescues a Drosophila model for pantothenate kinase-associated neurodegeneration. Proc Natl Acad Sci U S A 107: 6988–6993. 20351285 10.1073/pnas.0912105107 PMC2872433

[13] Kall L, Krogh A, Sonnhammer EL (2007) Advantages of combined transmembrane topology and signal peptide prediction-the Phobius web server. Nucleic Acids Res 35: W429–432. 17483518 10.1093/nar/gkm256 PMC1933244

[14] Dietzl G, Chen D, Schnorrer F, Su KC, Barinova Y, et al (2007) A genome-wide transgenic RNAi library for conditional gene inactivation in Drosophila. Nature 448: 151–156. 17625558 10.1038/nature05954

[15] Gregory A, Hayflick S (2013) Neurodegeneration with Brain Iron Accumulation Disorders Overview. In: Pagon RA, Bird TD, Dolan CR, Stephens K, Adam MP, editors. GeneReviews Seattle (WA): University of Washington, Seattle; 1993–2013. 23447832

[16] Wittmann CW, Wszolek MF, Shulman JM, Salvaterra PM, Lewis J, et al (2001) Tauopathy in Drosophila: neurodegeneration without neurofibrillary tangles. Science 293: 711–714. 11408621 10.1126/science.1062382

[17] Muhlig-Versen M, da Cruz AB, Tschape JA, Moser M, Buttner R, et al (2005) Loss of Swiss cheese/neuropathy target esterase activity causes disruption of phosphatidylcholine homeostasis and neuronal and glial death in adult Drosophila. J Neurosci 25: 2865–2873. 15772346 10.1523/JNEUROSCI.5097-04.2005 PMC1182176

[18] Kretzschmar D, Hasan G, Sharma S, Heisenberg M, Benzer S (1997) The swiss cheese mutant causes glial hyperwrapping and brain degeneration in Drosophila. J Neurosci 17: 7425–7432. 9295388 10.1523/JNEUROSCI.17-19-07425.1997 PMC6573436

[19] Jaiswal M, Sandoval H, Zhang K, Bayat V, Bellen HJ (2012) Probing mechanisms that underlie human neurodegenerative diseases in Drosophila. Annu Rev Genet 46: 371–396. 22974305 10.1146/annurev-genet-110711-155456 PMC3663445

[20] Auluck PK, Chan HY, Trojanowski JQ, Lee VM, Bonini NM (2002) Chaperone suppression of alpha-synuclein toxicity in a Drosophila model for Parkinson’s disease. Science 295: 865–868. 11823645 10.1126/science.1067389

[21] Cowan CM, Sealey MA, Quraishe S, Targett MT, Marcellus K, et al (2011) Modelling tauopathies in Drosophila: insights from the fruit fly. Int J Alzheimers Dis 2011: 598157. 22254145 10.4061/2011/598157 PMC3255107

[22] Soldano A, Okray Z, Janovska P, Tmejova K, Reynaud E, et al (2013) The Drosophila homologue of the amyloid precursor protein is a conserved modulator of Wnt PCP signaling. PLoS Biol 11: e1001562. 23690751 10.1371/journal.pbio.1001562 PMC3653798

[23] Jackson GR, Salecker I, Dong X, Yao X, Arnheim N, et al (1998) Polyglutamine-expanded human huntingtin transgenes induce degeneration of Drosophila photoreceptor neurons. Neuron 21: 633–642. 9768849 10.1016/s0896-6273(00)80573-5

[24] Benzer S (1967) Behavioral mutants of Drosophila isolated by countercurrent distribution. Proc Natl Acad Sci U S A 58: 1112–1119. 16578662 10.1073/pnas.58.3.1112 PMC335755

[25] Zhang H, Tan J, Reynolds E, Kuebler D, Faulhaber S, et al (2002) The Drosophila slamdance gene: a mutation in an aminopeptidase can cause seizure, paralysis and neuronal failure. Genetics 162: 1283–1299. 12454073 10.1093/genetics/162.3.1283 PMC1462322

