# Supplementary figures and images for: Impairment of Drosophila Orthologs of the Human Orphan Protein C19orf12 Induces Bang Sensitivity and Neurodegeneration
Source: PLoS One. 2014 Feb 21;9(2):e89439. doi: 10.1371/journal.pone.0089439 (PMC3931782; doi:10.1371/journal.pone.0089439)

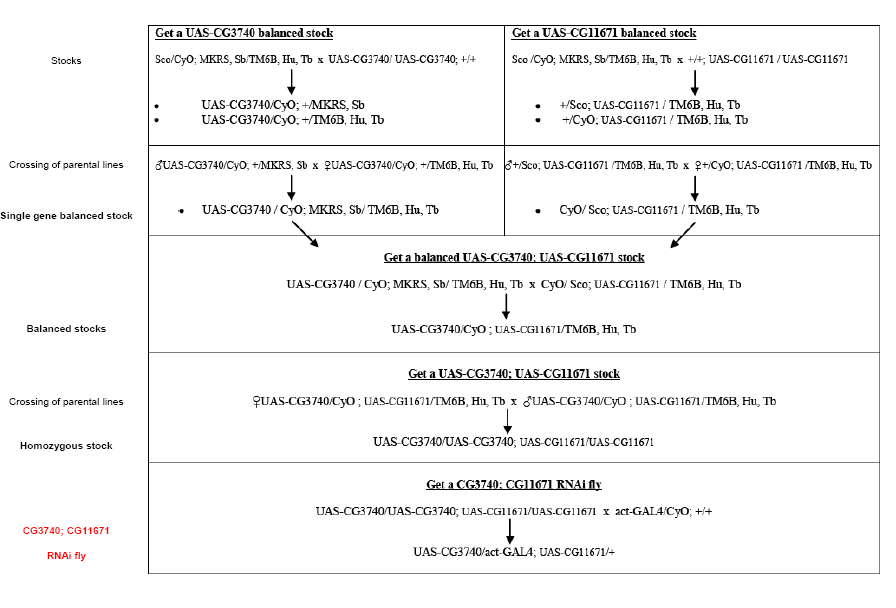

Supplement: Figure S1 — Generation of double RNAi Flies. UAS-CG3740 and UAS-CG11671 flies have been crossed respectively with the double balancer CyO/snaSco; TM6B/MKRS to block any recombination on the second and on the third chromosome. Hence selected parental lines have been crossed between each other in order to get the double balanced stock UAS-CG3740/CyO; UAS-CG11671/TM6. The homozygous stock UAS-CG3740/UAS-CG3740; UAS-CG11671/UAS-CG11671, produced by backcrossing balanced males and females, has been crossed with act-GAL4 driver line to get the double RNAi flies. (TIF) [file pone.0089439.s001.tif]

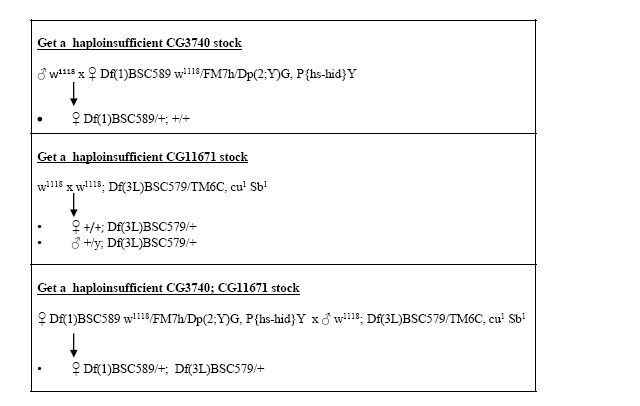

Supplement: Figure S2 — Generation of double heterozygous deletion Flies. Flies carrying deficiencies on chromosomes 1 (BL25423) and 3 (BL25413) have been crossed with wild type flies w1118 to generate single heterozygous deletion flies for CG3740 and CG11671. Then deficiency stocks have been crossed between each other in order to get the double heterozygous deleted stock Df(1)BSC589/+; Df(3L)BSC579/+. (TIF) [file pone.0089439.s002.tif]
