# Supplementary material for: Impairment of Drosophila Orthologs of the Human Orphan Protein C19orf12 Induces Bang Sensitivity and Neurodegeneration
Source: PLoS One. 2014 Feb 21;9(2):e89439. doi: 10.1371/journal.pone.0089439 (PMC3931782; doi:10.1371/journal.pone.0089439)
Supplement: Text S1 — Generation of act -GAL4/+ flies. Act5C-GAL4/CyO (BL4414) and CyO/snaSco (BL2555) flies, were backcrossed with w1118 flies for 6 generations to produce control flies isogenic with w1118 for chromosomes 1, 3, and 4. (DOCX) [file pone.0089439.s003.docx]

Text S1. Backcrossing scheme. Virgin act5C-GAL4 /CyO females were crossed with y^1^w^1118^ males. From the first progeny w^+mC^ (non-CyO) virgins, (because of the w^+mC^ in the act5C construct), were taken and crossed again with y^1^w^1118^ males. This was repeated for 6 generation and act5C-GAL4/+ males and females were collected.

In parallel virgin CyO/sna^Sco^ females were crossed with y^1^w^1118^ males. From the first progeny CyO bearing virgins were taken and crossed again with y^1^w^1118^ males. This was repeated for 6 generation and CyO/+ males and females were collected.

act5C-GAL4/+ females were crossed to CyO/+ males and viceversa and act5C-GAL4/CyO progeny was recovered and used as driver line.
